# Supposed Poisoning by Mercury

**Published:** 1830-10-01

**Authors:** 


### SUPPOSED POISONING BY MERCURY.

M. ORFILA has published a long paper on this subject in a recent number of the ARCHIVES, the proximate cause of which may be first succinctly stated.

On the 3d of July, 1829, a female named VILLOING, residing at St. Brisson, having then been ill for five or six days, was visited by Dr. Carron, at her own request. She complained of great oppression at the epigastrium — frequent inclination to vomit, and occasional discharges of bilious matters—full pulse—flushed face—eyes and countenance yellow. The husband said that the cause of his wife's illness was fatigue. Dr. C. ordered a grain of tartar-emetic by lavement, and desired the husband to report next day. A report was made that she was rather better, but still harrassed with sickness. He prescribed an opiate. Two days afterwards the doctor was summoned in great haste to the patient, but did not find her worse, according to appearances, and still considered the complaint as a bilious affection, reiterating the opiate medicine. He prognosticated a speedy recovery: but on the morning of the 7th July, he was astonished to learn that the patient had died the preceding evening. The vomitings, he was informed, had become extremely frequent for some hours before death. Several poisonous substances, as oxymuriate of mercury, arsenic, &c. were found in the house. The body had been buried, without any suspicion of poisoning; but was disinterred some days afterwards by orders of the Procureur du Roi. The examination of the body by Drs. Carron and Ballot, took place on the 22d July, 15 days after interment. Considerable decomposition had taken place, and the most horrible effluvium issued from the body. The thorax, the heart and lungs offered nothing remarkable. The internal membrane of the œsophagus was injected, but neither softened nor ulcerated. On opening the abdomen, the dissectors were astonished to perceive that the abdominal viscera appeared to be those of a corpse recently deprived of life. No effusion, no adhesion. There was a considerable quantity of air in the intestines; but the sides of the stomach were in close contact. On closer examination, two perforations were found in the stomach at its inferior and anterior part, together with several discoloured spots, both on the exterior of the stomach and of the intestines. The liver was greatly enlarged, and its surface emphysematous. The pancreas and spleen, although of a dark color, were not altered in texture by putrefaction. The same with the kidneys. The stomach was very large, and a patch of intense redness, two inches in extent, was visible on the mucous membrane of the smaller curvature, and the two perforations abovementioned, the external apertures of which were wider than the internal. The mucous membrane of the organ was intensely red about the cardiac orifice, and this redness spread along the lesser curvature. The great cul de sac was also the seat of red arborization, in which were erosions. The internal orifices of the perforations were clean cuts, as if made by a circular chissel, and without any surrounding redness. There was nothing unusual in the appearance of the pylorus. In the stomach, duodenum,—in short, throughout the whole tube were found numerous globules of mercury, and about two drachms were collected pure. The membranes themselves were covered with a kind of mercurial dew (rosée mercurielle) formed by globules infinitely divided. Numerous experiments made on the contents and on the tissues of the stomach, intestines, and other organs of the body, could not detect any trace of poisonous substance, beyond the metallic mercury abovementioned.

The two physicians made the following remark. As there is no other way of proving the administration of corrosive sublimate, after it has combined with the tissues and formed submuriate of mercury, than by reproducing the metal, and as crude was already found in the stomach and bowels, such revivication of the metal would not afford any proof of poison, since it might be said that the metallic mercury recovered from the tissues, was only the metallic mercury that had penetrated into them previously. Nevertheless they thought such a reproduction would afford suspicions of poison having been taken.

M. Orfila then set about making two series of experiments—the *first* by exhibiting to dogs the oxymuriate of mercury, with the view of obtaining the crude metal from the tissues with which it had combined—the *second*, by exhibiting the same oxymuriate, along with substances that are known to be capable of decomposing it in our laboratories. In respect to the *first* series:—If thirty or forty grains of sublimate are given to a dog, he is destroyed in a few hours—from four to ten or twelve. If the body be buried for three or four months, then disinterred, and examined, no crude mercury can be discovered. But if the tissues are exposed to the action of potassa at a white heat, crude mercury will be volatilized from the state of calomel in which it had been transformed by the combination with animal matters. This proves, therefore, that the decomposition of sublimate in the body does not present globules of mercury but submuriate of quicksilver.

*Second series.* Corrosive sublimate, dissolved in water, is decomposed, so as to furnish metallic mercury, by iron, copper, zinc, arsenic, and phosphorus. Albumen, gelatine, alcohol, or oil have not this effect.

By numerous experiments and reasonings, however, which we have not room for here, M. Orfila comes to the conclusion that there was no proof whatever afforded that any of the salts of mercury had been swallowed by the deceased VILLOING. He stated that the probability was that the post-mortem appearances were the result of disease, (inflammation) and that crude mercury had probably been administered under the popular notion that obstructions are cleared away, and pains alleviated by that metal.

